# Effective advocacy strategies for influencing government nutrition policy: a conceptual model

**DOI:** 10.1186/s12966-018-0716-y

**Published:** 2018-08-31

**Authors:** Katherine Cullerton, Timothy Donnet, Amanda Lee, Danielle Gallegos

**Affiliations:** 1 School of Public Health, University of Queensland, Wyndham St, Herston, QLD 4006 Australia; 2 Queensland University of Technology, QLD, Brisbane, Australia; 3 The Australian Prevention Partnership Centre, The Sax Institute, Ultimo, NSW Australia; 4 School of Exercise and Nutrition Sciences, Queensland University of Technology, QLD, Kelvin Grove, Australia

**Keywords:** Policy, Public health, Nutrition, Advocacy, Policy making, Political will

## Abstract

Influencing public policy change can be difficult and complex, particularly for those with limited power and resources. For any one issue there may be several groups, including the commercial sector and public health advocates advocating from different policy perspectives. However, much of the public health advocacy literature and tools available for those wanting to improve their practice is based on research from one specific perspective of an issue. This approach deprives advocates of potential insight into the most effective levers for this complex and difficult process. To provide a more comprehensive insight into effective levers for influencing public health policy change, a conceptual model for poorly-resourced advocates was developed. The model was developed through the integration and synthesis of policy process and network theories with the results from three studies conducted previously by the authors: a systematic literature review; a social network analysis of influential actors in Australian nutrition policy; plus in-depth interviews with a sample of these actors who had diverse perspectives on influencing nutrition policy. Through understanding the key steps in this model advocates will be better equipped to increase political and public will, and affect positive policy change.

## Background

Influencing public policy change can be difficult and complex, particularly for those with limited power and resources. One of the key difficulties is that the development of public policy is rarely a linear process [1]. It is constructed through complex interactions and negotiations amongst a range of stakeholders, including politicians, interest groups, advisers, bureaucrats, and a range of other actors [2]. Many factors impact on the likelihood of policy change occurring. Gaining the support of the public is one important factor in policy change, however, ensuring you have political will is essential [3]. For those outside policymaking circles, particularly from resource-poor organisations, influencing the policy process can seem an impossible task. However, there are strategies that these individuals or organisations can adopt to increase their influence. Understanding and applying these strategies, but also understanding the factors that may detract from them, can change the power dynamic between policymakers and public health advocates and increase the likelihood of influencing the policymaking process.

The process of undertaking active interventions with the explicit goal of influencing government policy is known as advocacy [4]. For public health nutrition policy there is often several groups, including the commercial sector, advocating from different policy perspectives. However, much of the public health advocacy literature and tools available for those wanting to improve their practice are based on research from one specific perspective of an issue [5–8], that is, policymakers (senior politicians responsible for a portfolio or very senior bureaucrats), more junior government bureaucrats and/or health advocates have reported what has previously worked for them. Alternatively it may be an individual promoting the successful strategies they have used, which they hope others will be able to utilise [9, 10]. However, by only examining success stories from one perspective of the issue, advocates deprive themselves of potential insight into the most effective levers for this complex and difficult process.

At a theoretical level, there are policy process theories available from political science scholars who have investigated agenda-setting and policy change over decades (see Table 1). While these theories are extremely helpful in understanding the broader factors that explain the policymaking process, in particular agenda-setting and policy change [11–13], they are mostly used retrospectively to examine policy change and do not provide the direct, practical guidance many health advocates on the ground require to increase their influence in policymaking [14].

**Table 1:** Summary of theories used in the development of the conceptual model

	Advocacy Coalition Framework [11]	Multiple Streams Theory [12]	Punctuated Equilibrium Theory [13]	Strength of Weak Ties [19]
Summary of theory	Policymaking is characterised by the interaction of advocacy coalitions within a policy system. Belief systems guide choices and actions. Alignment and activity of coalitions can drive change.	Policymaking is composed of three streams: problem; policy: and politics. When these streams come together during open policy windows, policy change is likely to occur. Policy entrepreneurs play a crucial role in this process.	Policymaking is characterised by long periods of incremental change punctuated by brief periods of major change. Policy image (framing) and public mobilisation play a central role in aiding policy change.	Possession of links to actors beyond one’s immediate close knit cluster can greatly increase opportunities for new or distinct information. Access to this information can provide new insights enabling advocates to better influence policymaking.

By integrating key policy process theories with our previous research on power and influence in public health nutrition policymaking, we have developed a conceptual model to help guide stakeholders who are poorly-resourced either through lack of funding, skills and/or time, to more effectively influence the policymaking process. Public health nutrition was chosen as the focus for this model because there has been limited national policy action occurring in the field of public health nutrition in Australia for the past decade [15, 16]. This limited policy action can be partly explained through the power and influence of the food industry but also through the limited advocacy skills, knowledge and resources that nutrition professionals, be that practitioners or academics, possess [3, 16]. Through understanding the key steps of the conceptual model we have developed advocates will be better equipped to increase political and public will, which may better facilitate positive public health nutrition policy change.

## Methods

The conceptual model described in this paper was developed through the integration and synthesis of results from three studies published previously by the authors along with policy process and network theory [3, 16–18]. The purpose of these studies was to explore the factors influencing public health nutrition policymaking in Australia. Methods across the three studies included a systematic literature review identifying the barriers and enablers to public health nutrition policy change [3], social network analysis with a focus on network structures, clusters and normalised measures of centrality dispersion [16, 18], and in-depth semi-structured interviews with key nutrition policy influencers (*n* = 37) including health advocates, food industry senior executives and politicians, examining the factors enabling nutrition policy change [17]. Incorporated into the design and the analysis of each of these studies was policy process theory [11–13] and network analysis theory (see Table 1) [19, 20].

Detailed methods for each primary study have been reported in their respective publications, however, they are briefly outlined in Table 2 for reference.

**Table 2:** Summary of study designs and findings used to inform conceptual model

Type of study	Method	Key findings
Systematic literature review [3]	This systematic review identified and synthesized the enablers and barriers to public policy change within the field of nutrition from peer-reviewed literature. Sixty three studies examining policymaking in public health nutrition in high income-democratic countries were included. An interpretive synthesis, involving induction and interpretation to identify key themes, was undertaken.	• Political will is required for policy change • Public will is an enabler, but not essential for change • Health professionals find it difficult to influence nutrition policy change • Barriers and enablers do exist that may be of use to health professionals. These include: pressure from industry, neoliberal ideology, use of emotions and values, and being visible.
Network analysis [16, 18]	Social network analysis techniques were used to explore the capacity of different individuals and interest groups to influence nutrition policymaking networks in Australia. Four rounds of data collection was undertaken and the capacity of individual actors and occupational categories e.g. food industry, nutrition academic, to influence policy decision-makers were analysed. Cluster analysis, and two measures of influence: path distance of actors from decision-makers and betweenness centrality, were also undertaken.	• The food industry has the greatest capacity to influence nutrition policy in Australia compared to all other professional categories. • Nutrition professionals are far removed from key policy decision-makers, with limited strategic relationships. • There are two key brokers, a general health professional from a non-government organisation and a nutrition academic, in the network that both play different brokerage roles.
In-depth interviews [17]	Thirty seven nutrition policy decision-makers and key influencers were purposively selected to participate in semi-structured, in-depth interviews which examined the key barriers and enablers to nutrition policy change. Participants were chosen based on their ability to represent views from different ‘sides’ of the issue and obtain maximum diversity. They included health advocates, food industry senior executives, government policy officers, politicians and academics. Data analysis was undertaken using an adapted version of the Framework Method which included systematic coding, analysis and synthesis of the data to develop themes and categories [61].	• Influencing nutrition policy is a complex and dynamic process with a series of inter-related barriers and enablers. • The strategy of investing in relationships underpinned the whole process. • Crucial contextual factors (pressurised, risk-averse environment; system of governance; neoliberal environment; and the democratisation of knowledge) that also impact on nutrition policymaking in Australia were identified.

### Development of conceptual model

To develop the conceptual model, the question “how can resource-poor organisations increase their influence in the nutrition policymaking process?” was asked and answered by comparing and contrasting the themes from the three studies. The themes identified in the three studies had been previously derived through a mix of inductive and deductive analysis. Key concepts from policy process theories and network theories, in particular Multiple Streams Theory [12], Advocacy Coalition Framework [21], Punctuated Equilibrium theory [13], and the Strength of Weak Ties [19] theory had been used as deductive codes when coding the in-depth interview data and the systematic literature review.

The themes from each study were prioritised to determine which themes/factors were central to answering the research question. This process was underpinned by a pragmatic research paradigm, which focuses on bringing together multiple sources of knowledge with the goal of finding workable solutions [22, 23].

In addition, policy process and network theories were also used as interpretive lenses to aid overall integration and synthesis of the three studies by drawing our attention to different aspects of the data. This was followed by concept mapping the relationships between the identified themes and the research question. Feedback from the research team resulted in multiple iterations of the map until all agreed that the explanatory graphical model answered our research question [24]. Using multiple sources of data and methods enabled us to continuously consider and incorporate diverse viewpoints around influencing the policymaking process into the conceptual model [25]. QUT University Human Research Ethics Committee provided ethical approval (Approval Number 1400000857).

## Results and discussion

Key themes from three studies on power and influence in public health nutrition policy were identified and synthesised into a conceptual model to increase the influence of advocates in nutrition policymaking. The convergence of the key themes from the three studies and their relationship to the elements of the conceptual model is presented in Table 3. As can be seen in this table there is inter-relatedness between the themes of the different studies. Unsurprisingly, the in-depth interviews provided greater depth and insight into influencing political will and the policymaking process.

**Table 3:** Summary of themes identified in research against elements of conceptual model

	Key themes from studies		
Components of model	Systematic literature RV	Network Analysis	In-depth interviews
Contextual factors			
Neoliberal environment	‘Understand the policymaking process’ ‘The rise of neoliberal ideology’		‘Understand the policymaking process’ ‘Power and influence of food industry’ ‘Appeal to beliefs’ ‘Abdication of responsibility’ ‘Evidence is only one factor’
Pressurised, risk-averse environment	‘Understand the policymaking process’ ‘Pressure from industry’		‘Understand the policymaking process’ ‘Evidence is only one factor’ ‘Competing for attention of decision-makers’ ‘Lack of public will’ ‘Complex, multifaceted problem’
System of governance	‘Understand the policymaking process’ ‘Government silos’		‘Understand the policymaking process’ ‘Be alert for policy window’ ‘Priority of other portfolios’ ‘Evidence is only one factor’ ‘Lack of public will’
Democratisation of knowledge			‘Complex, multifaceted problem’ ‘Evidence is only one factor’ ‘Competing for attention of decision-makers’ ‘Appeal to beliefs’
Enablers			
Invest in relationships/gather intelligence	‘Build relationships with key stakeholders’ ‘Understand the policymaking process’ ‘Be visible’	‘Invest in diverse & strategic relationships’	‘Invest in relationships’ ‘Understand the policymaking process’ ‘Be alert for policy window’ ‘Credibility’ ‘Trust’
Develop clear, unified solution	‘Develop a well thought-through solution’		‘Provide an attractive solution’ ‘Represent many voices’
Engage or develop skills of a policy entrepreneur	‘Engage a policy entrepreneur or develop skills if advocates’ ‘Understand the policymaking process’	‘Have more than one policy broker/entrepreneur’	‘Credibility’ ‘Invest in relationships’ ‘Be alert for policy window’ ‘Play the long game’ ‘Issue/name top of mind’
Engage policy champion	‘Be visible’ ‘Understand the policymaking process’	‘Policy brokers/entrepreneur may not be as powerful as previously thought’	‘Power and status’ ‘Trust’ ‘Respect’ ‘Credibility’ ‘Appeal to beliefs’
Reframe issues appealing to values and beliefs	‘Use emotions and values’		‘Provide an attractive solution’ ‘Appeal to beliefs’
Amplify frame	‘Be visible’		‘Invest in relationships’ ‘Able to create noise’ ‘Build/mobilise coalitions’
Increase public will	‘Increase public will’ ‘Be visible’		‘Invest in relationships’ ‘Able to create noise’ ‘Build/mobilise coalitions’

**Fig. 1:**
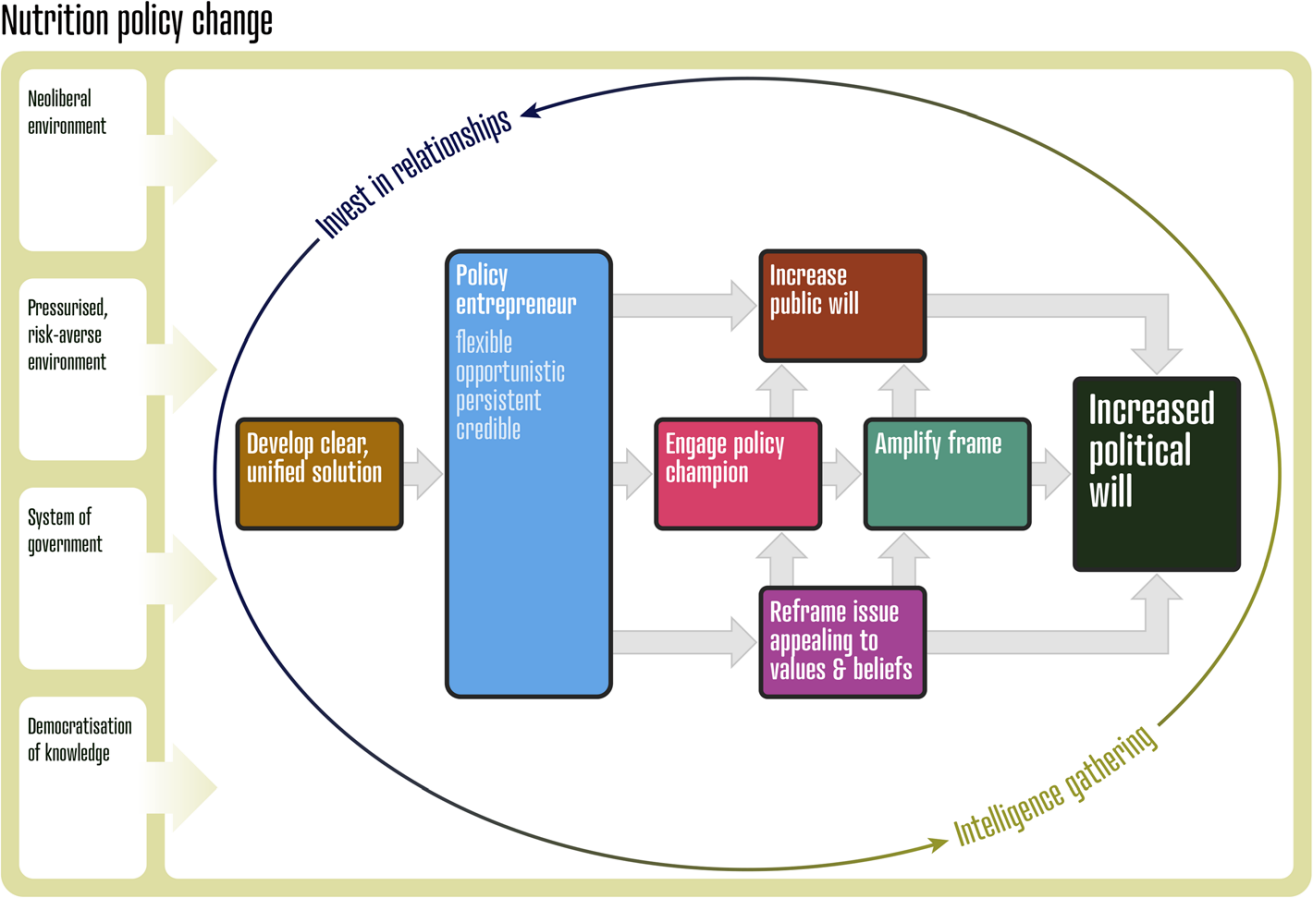
A conceptual model for influencing government nutrition policy

The elements of the conceptual model (Fig. 1) will now be examined and discussed in more detail with illustrative quotes from the in-depth interviews of 37 key nutrition policy influencers [17]. It is important to note that this model does not encapsulate all of the findings from the three studies or all facets of influencing the policymaking process; rather, the model serves as a practical, heuristic model specifically for poorly-resourced individuals or organisations. These individuals or organisations are characterised by having limited time, skills and/or finances to undertake advocacy. By incorporating the original studies’ findings [17], the model outlines pragmatic strategies that can be applied to increase policy actors’ influence in public health nutrition policymaking at a national level. The strategies may appear consecutive, however their implementation should be more iterative, with several steps occurring simultaneously if possible. Furthermore, the strategies should be refined continuously supported by frequent intelligence gathering. For organisations unable to resource all steps of the model, we suggest investing in the essential strategies of [17]:

- investing in relationships
- gathering intelligence
- developing a clear, unified solution, and
- employing or developing the skills/traits of a policy entrepreneur.

The findings from our previous three studies suggest that for the remaining strategies, assessing the actor’s strengths and resources is important to ensure the selection of remaining strategies aligns with their capacities and capabilities.

### Contextual factors

Policy change can occur when a ‘policy window’ - or an opportunity for change - opens [12]. This can be an infrequent occurrence and policy windows often open and close before anyone has the chance to exploit them [12, 26]. Advocates need to understand the context around these opportunities for change before undertaking advocacy, as the wider political environment should inform an advocate’s selection of influencing strategies [27]. Four important contextual factors were identified as part of the conceptual model: the neoliberal environment; a pressurised and risk-averse environment; the inherent system of governance; and democratisation of knowledge. These factors should be kept in mind by others seeking to adopt or translate the conceptual model to their own context.

Neoliberalism is an ideology characterised by market deregulation, privatisation of the public sector, and the promotion of individual responsibility [28]. It increasingly influences government decisions around recognising issues and their subsequent intervention [29]. Both the systematic literature review [3] and in-depth interviews [17] highlighted that advocates need to recognise and work within the constraints of neoliberal ideology, particularly with political parties who have greater allegiance to neoliberal ideology:

*“There’s this reluctance on the part of government to get involved in this area … because you revert to this notion of self-responsibility and how much do you intervene.”* (State Politician) [17].

If such a party is in power, our findings highlighted that advocates should recognise that certain policy options may never be endorsed. Instead our findings, alongside the policy literature, suggest that advocates be patient and wait for political change or undertake a process of venue shopping; that is, investigate whether other departments or jurisdictions are interested in your problem and solution [12, 13].

Another contextual factor highlighted by our studies and the policy process literature is that advocates should understand that policymakers work in pressurised, risk-averse environs [12, 13], and often lack the time to consider every advocate’s concern with equal attention.

*“Remember information overload affects us all. It certainly affects bureaucrats and ministerial staffers, they just get shit loads…They’ve also got 50 million people in their faces all wanting something.”* (Food Industry 7) [17].

This environment requires advocates to adopt strategies that can resonate quickly with policymakers - although it can also mean that policy change may take a long time, possibly ten years or more [11, 30].

Understanding the system of governance in the advocate’s country, particularly with respect to the formal and informal rules of policymaking and who has power over these rules is crucial to effective advocacy. This requires gathering intelligence about the system, its key influences, and spaces where power is concentrated in the policy network:

*“The first rule is always understand who actually makes the decision…On what basis do they make the decision? Who are they genuinely influenced by and how do you manage those processes?”* (Lobbyist) [17].

Adding to these three areas we found an issue ubiquitous to nutrition policy; the increasing democratisation of knowledge. Our in-depth interviews revealed that the ease of access to scientific evidence has dramatically increased with the internet, effectively transforming lay persons into ‘experts’ particularly in the field of nutrition. The general public’s active interest in health and nutrition has led to increased competition in the nutrition policymaking space effectively diluting the voice of nutrition scientists and professionals in policymaking.

“*Everybody’s an expert in nutrition…So it’s hard for scientists in the area to get traction because everyone’s opinion seems to be given equal weigh on the web”*. (Public Health Academic 2) [17].

While the internet is part of the reason behind this rise in non-traditional ‘experts’, interview participants also highlighted that there was general mistrust of the information coming from some professional nutrition associations due to partnerships with the food industry [17]. Furthermore, participants identified that nutrition professionals displayed a limited understanding of the lived experience of the general public in relation to their nutrition-related problems and solutions. This decreases the perceived legitimacy, and hence influence, of nutrition professionals with the general public and policymakers [31, 32].

While these contextual issues can present barriers to policy change, our research suggests there are a range of strategies available that can increase the influence of advocates in the policy process; these will now be discussed.

#### Invest in relationships strategically

The gathering and presentation of scientific evidence is often prioritised by nutrition advocates in an effort to influence policy change [1, 3, 33]. Our studies plus others [3, 33] have found this strategy alone has limited influence on policymaking:

*“You could have all the evidence in the world and it won’t get you action. And sometimes you can get action without any evidence. They're really important factors to think about at the political level.”* (Federal Bureaucrat 3) [17]

Instead, our findings demonstrated that investing in relationships strategically, underpinned the process for influencing policy change [3, 16–18]. By prioritising ‘investing in relationships’, advocates are able to: develop trust and increase credibility with stakeholders which may lead to coalitions or alliances; identify prospective policy champions; gather intelligence on policy opportunities and risks plus the values and beliefs of decision-makers and key influencers; and gain an understanding of the arguments of opponents. Our network analysis demonstrated that the food industry currently hold a striking advantage in their ability to influence nutrition policy in Australia through the high number of direct relationships with decision-makers [16]. In contrast, nutrition professionals lack these connections and strategic approach, and instead concentrate on building relationships with other nutrition professionals. Consistent with network theory [19] our findings suggest that for nutrition professionals to increase their influence they must invest in a diverse range of relationships with policy actors across the policy spectrum. Importantly, relationships cannot be developed with everyone, hence the requirement to be strategic and target those individuals with the greatest potential for quality intelligence and influence [34]. This is turn links back to gathering intelligence to determine who those individuals are.

A deep understanding of the policymaking environment is essential for formulating an advocacy strategy as it provides insight into policy opportunities and barriers as well who is influential in the policymaking process [14]. Understanding these factors allows a more nuanced and effective advocacy strategy to be developed. This knowledge can be gained by reading literature on the topic, experience in the policy sector, or through developing relationships and gathering intelligence with those involved in the policymaking process. However, building these relationships and gaining knowledge requires time and continual investment in the relationships:

*“The organisations that are influential to me are the organisations that get in touch with me… I know who they are, I trust them, the information they have, you have that constant relationship…so you think of them at the top of the pile.”* (Federal Politician) [17]

The formation of coalitions or alliances can also result from investing in relationships. This strategy for influence is supported by policy process theory [11] and our systematic review and in-depth interviews highlighted that forming coalitions or alliances was a particularly useful strategy for poorly-resourced organisations. Several interviewees explained that when a variety of organisations are in agreement on an issue this signals to decision-makers that the issue has considerable support and increases your voice on the issue:

*“The more* [organisations] *you can collect together and say I want this, the stronger the message to the government.”* (Federal Bureaucrat 1) [17]

Furthermore, our findings highlighted that forming an alliance or coalition allows poorly–resourced organisations to pool resources and coordinate advocacy action [14]. However, as seen in the network analysis, there is a propensity for nutrition coalitions to involve homogenous members [16]. This can result in good internal communication and networking, but may hinder members gathering new intelligence and engaging others in new ideas [19, 20]. For better outcomes, a strategic approach should be taken to ensure alliances or coalitions cover a wide range of interests, skills and personal contacts.

The in-depth interviews revealed another benefit of strategically investing in relationships, which was intelligence gathering on the ‘opposition’ and their arguments:

*“I have always tried to engage with as many stakeholders as I can. I try to make sure that we can understand everybody else's position and then at the same time communicate what our position is and our constraints and our own issues.”* (Food Industry 5) [17]

Gathering this intelligence provides advocates with a deeper understanding of competing points of view, enabling the development of strong counter arguments [35, 36]. Notably, relationships with opponents need to be carefully navigated by advocates to ensure their credibility, as seen by policymakers and the general public, remains intact [37].

The remaining elements of the model will now be discussed, however it is important to remember investing in relationships and gathering intelligence underpins every element in this model [17].

#### Develop a clear, unified solution

Gaining the attention of policymakers is difficult with a complex issue due to the bounded rationality of policymakers [13]. Bounded rationality encapsulates the idea that humans have limited resources to process stimuli, consequently decision-making often occurs using cognitive shortcuts rather than comprehensive analysis [38]. Our studies found that to overcome this naturally occurring process, the problem and solution being offered to policymakers requires simplification [3, 17]. This can be challenging in the field of public health nutrition as most issues are inherently complex and often there is a limited agreement on what should take priority. However, the participants in our study and the evidence to date show that when an issue is presented as complex and requiring complex solutions, policy change is unlikely to occur as it is considered too hard [39, 40]. This may mean that small incremental policy actions are favoured, as opposed to policies that propose radical changes to existing systems. Consequently, advocates need to recognise that achieving policy action may take considerable time and/or carefully crafted implementation plans for each phase of change.

The requirement for clear, unified solutions raises the wider issue of competing agendas within the nutrition policy space. This issue was found in the systematic review and raised by several of the in-depth interview participants:

*“One of the really key things generally is you've got a much better chance at getting action when it's clear what the action should be. In the nutrition case, it's not at all clear what the action should be.”* (Federal Bureaucrat 3) [17]

Nutrition policy scholars have affirmed that fragmented advocacy in the field of nutrition can deter policy change and result in ‘attention fatigue’ in policymakers [17, 40]. Similarly, other studies [27] have demonstrated that when policymakers see the demands of individual interest groups not supported by others, they will avoid advocating for the issue. This places greater emphasis on the need for strategic alliances and networks of stakeholders to coordinate similar policy agendas into a coherent message for decision makers to interpret.

#### Employ or develop the skills of a policy entrepreneur

The limited resources, time and skills of advocates were identified in both the in-depth interviews and the systematic review as significant barriers to effectively influencing nutrition policy change. Organisations who invest in developing ‘entrepreneurial’ skills of a dedicated staff member, or who employ a ‘policy entrepreneur’, can gain considerable influence in the policymaking process [12, 17]. Policy entrepreneurs are typically described in the policy literature as individuals who “wait in and around government with their solutions at hand, waiting for problems to float by to which they can attach their solutions, waiting for a development in the political stream they can use to their advantage.” [12]

Frequently the terms ‘policy entrepreneur’ and ‘policy champion’ have been used interchangeably in nutrition policy literature with no clear distinction between the two. However, in our study it was clear that the roles were distinct in one particular way; the power and status they hold [17]. We found that a policy entrepreneur best describes the role of a successful advocate – they could be a bureaucrat or a politician operating as an ‘insider’, or they might be an ‘outsider’ from a non-government organisation, academia or even a motivated member of the public. While policy entrepreneurs have varying levels of power and status, we found the very skilled policy entrepreneurs rely heavily on the ‘art’ of advocacy and are defined by their opportunistic, flexible, persistent, and credible nature and the priority they give to investing in relationships.

*“I am the type of guy who gets along with people …I’m thoughtful, persistent, all of those sorts of qualities.”* (Lobbyist) [17]

*“I’m reasonably good at working out where the win-win is. You know, trying to work out what the government needs, and what we need, and what the solution might be for that.”*(Food Industry 7) [17]

Some of these entrepreneurial skills are inherent; however some can be learnt. In particular, investing in relationships and gathering intelligence which allows policy entrepreneurs to identify policy opportunities and leverage points for decision-makers as well as understand the strategies of their opposition [41].

#### Secure a policy champion

In contrast to policy entrepreneurs, it was apparent in our studies that policy champions may not have or need the characteristics of successful policy entrepreneurs; instead their influence results from the high status and power they possess [17]. Often they will be an ‘insider’ in a position of formal authority, for example a very senior bureaucrat or a powerful politician. Accordingly, a Cabinet minister who takes on the role of a policy champion is perfectly positioned to take an issue into the Cabinet room and advocate for it:

*“Clearly the treasurer has greater sway than the health minister or the agriculture or industry minister but if any of those ministers come up with a convincing argument about something ….then you can convince either the majority of your ministry or Cabinet or party room or the leader and affect policy in that way.”* (Political Advisor 2) [17]

Alternatively, if a Cabinet minister cannot be secured as a policy champion, other Members of Parliament or Senators are also in a powerful position as they can directly advocate to their colleagues in Cabinet through their professional and personal relationships:

*“The best way to have an impact on that process is to get in the ear of someone who actually cares about what you’re talking about and get them to be a champion of your issue... We’ve got 54 or 55 caucus members… one of them is going to be interested in your issue and have time for it...”* (Federal Politician) [17]

We also found that there are policy champions who are *not* ‘insiders’. Usually these powerful individuals are from large food industry organisations who are able to command or demand an audience with decision-makers:

*“People that come on a large industry base, who are big employers…governments have to listen to them. [CEO of large food company] can bang on any door and get access to ministers at any time and so can [CEO of food retailer], incredibly influential, an absolute operator…People like him are able to knock on any doors.”* (Federal Bureaucrat 2) [17]

Securing a policy champion whether they were an ‘insider’ or an ‘outsider’ was seen to result in increased political will for an issue [17]. While this knowledge is valuable it is also presents challenges as securing a policy champion will be difficult for most advocates. Our findings suggest the most efficient way an advocate can secure a policy champion is to interrogate their networks for possible personal connections. This strategy can be broadened by mobilising alliance members and/or members of professional associations to specifically target their local Member of Parliament (MP) to become a champion, or to seek their recommendation for alternative MP’s who may be interested in the issue.

#### Reframe the issue to appeal to values and beliefs

In order for evidence around nutrition-related problems and solutions to be considered by policymakers, the issue must be framed to appeal to them and if possible the general public [11, 13, 42]. Frames are cognitive shortcuts that everyone uses to understand complex information more efficiently [14]. This occurs by selecting and emphasising attributes that communicate why an issue might be a problem, who is responsible for it, and what should be done about it [43]. The most effective frames appeal to shared societal values that resonate with individuals and in turn can motivate them to act [42]. Determining the most effective frame to use requires gathering intelligence on the values of the target audience [44]. Once these values are known, the problem and solution can be framed effectively to ensure it resonates with the target audience:

*“It’s deliberately opportunistic that we pick on the marketing of junk food for children, because if you just talk about the marketing of junk food, we're not going to get the same kind of results.”* (NGO General Health 4) [17]

Successful frames used previously include: protecting the health of children; truth and honesty; fairness and social justice; and highlighting potential economic and social losses related to policy inaction [45–47]. Frames highlighting ‘local’ issues have also been shown to increase an audience’s connection and solidarity with an issue [48].

#### Amplify the frame

To stand out above the ‘noise’ surrounding policymakers and the general public, we found that advocates need to amplify their frame. Amplification is intended to ensure the issue at hand and/or the advocate is clearly heard and becomes top-of-mind for policymakers and the general public. However, this step can be challenging as there many vested interests continually attempting to influence nutrition policy [16]. To overcome this competition and effectively amplify the frame, our studies highlighted a number of practical strategies that can be undertaken. The most common strategy to use the media, although engaging the media and then ensuring they report on the issue using the new frame is often difficult [13, 49, 50].

*“It’s always a positive strategy… If the media is drawing attention to them, if it’s an issue that the public is interested in and they have to focus on, then, of course, it’s important, but it can be difficult to get the media interested.”* (Political Advisor 2) [17]

Another strategy to amplify the frame is to identify individuals who are strategically placed within the nutrition policy network to advocate for the issue [18]. The policy champion will often be best placed to undertake this process internally. However, we found it was crucial that amplification efforts do not rely solely on one well-connected individual as this limits the effectiveness of amplification and can make the advocacy network vulnerable [51]. This was seen in our network analysis of nutrition policy in Australia [18]. To increase the chance of success, identify as many individuals as possible who can uniformly advocate and amplify the frame in a more coordinated way. Ideally this would result in simultaneous frame amplification targeting all Members of Parliament and influential bureaucrats. Large membership associations are at an advantage with this strategy as they are able to mobilise substantial numbers en masse. Alternatively, utilising members of coalitions or alliances increases the range and depth of options for frame amplification.

A final strategy to further amplify the frame involves advocates partnering with a citizen personally affected by the issue at hand to present their story to decision-makers and/or the general public [17]. Our findings and the literature highlight the powerful effect that personal stories of constituents have on politicians [52, 53]. This occurs because humans are able to cognitively process narratives or stories more efficiently than hard data or statistics [54]. These narratives will usually evoke emotion, making the information more memorable and more dominant in cognitive processing [53]. Our research suggests once this emotional connection has been made, scientific evidence regarding the problem and solution can then be presented.

#### Increase public will

Our in-depth interview participants considered gaining the support of the public for the issue at hand and demonstrating this level of support to policymakers was crucial for influencing policy change [17]. This strategy was supported in the systematic review which highlighted that decision-makers respond favourably to issues that have the support of their electorates, public officials and interest groups [50, 55–57]. Conversely, failing to demonstrate public support for a policy issue was identified as a key factor for a lack of policymakers’ support:

*“Some of the things that you learn very quickly in this political game, and I’ve come from no political background either, is unfortunately, a lot of decisions are made on how many votes they’ll receive for it.”* (Senator) [17]

Methods for building public will include several of the steps already discussed: using an effective frame, amplifying it, and investing in personal relationships – particularly with community groups. Importantly, a key finding from our in-depth interviews was ensuring the advocate or advocacy organisation is perceived to have credibility and is trusted. If an individual or organisation is believed to be trustworthy, it is more likely that the public will consider information from that individual or organisation to be truthful [58]. One factor shown to decrease credibility and trust in advocates or their organisation was a perception that they were associated with a commercial food company, in particular those which sell ‘unhealthy products’ [37, 59]:

*“With academics, they might do some fantastic research but if it was partially financed by Kellogg’s or something, people won’t believe it…Any of those organisations taking money from the ‘other side’…, it does weaken their credibility.”* (Federal Bureaucrat 4) [17]

Another factor leading to decreased credibility was the perception that the ‘nutrition message’ is always changing [17]. Ensuring nutrition associations and organisations present a unified public voice on nutrition matters will help to address this issue. However, negotiating a unified consensus and prioritisation of issues within the field of nutrition is particularly challenging as there are many interests at play.

#### Strengths and limitations

By gaining multiple perspectives around influencing nutrition policy we believe we were able to gain a richer and more in-depth understanding of the issue at hand [60]. The model developed for this paper was the culmination of several different studies using different methods to measure and interpret the same phenomenon. Using multiple methods overcomes the weaknesses of an individual method and increases validity [60]. The findings of our three studies were integrated with existing theories related to influencing policy change ensuring the conceptual model provides advocates with clear, detailed strategies that are theory-based but also grounded in the practical reality of the policy setting.

The development of this conceptual model is not without limitations. In developing a conceptual model that was realistic but also simple to use, not all factors that influence political will and policymaking have been included. Instead we have attempted to highlight the strategies that our evidence suggests are most important for poorly-resourced organisations to undertake. Another limitation is the studies used to inform the development of this model were from Australia or other high income, democratic countries and therefore the model may be limited to use in these countries. We acknowledge we may have missed important insight from the literature from low and middle-income countries. We encourage others to test our proposed model for increasing political will around nutrition policy change, particularly in different health or social service fields as well as different countries to determine whether it is normative. Undertaking this research would provide empirical analysis of whether this conceptual model is applicable in different settings.

## Conclusion

This study represents the synthesis of three different studies together with existing theoretical constructs into a conceptual model designed to be used by poorly-resourced organisations in high-income, democratic countries to increase their influence in public health nutrition policymaking. This conceptual model is intended to inform and guide the way advocates understand nutrition policymaking in order to increase their influence over it. No individual strategy will deliver results on its own. Similarly, undertaking all of the documented strategies may not result in policy action, as policymaking is a dynamic and complex process. However, our research and the literature highlight that by undertaking as many of these strategies as resources permit, and the more coordinated the approach, the more likely advocates will be to influence positive policy change.
